# Genome Sequence of Listeria innocua Strain MEZLIS26, Isolated from a Goat in South Africa

**DOI:** 10.1128/MRA.00991-19

**Published:** 2019-10-31

**Authors:** Mohamed E. El Zowalaty, Rachel A. Hickman, Alexandra Moura, Marc Lecuit, Oliver T. Zishiri, Noelle Noyes, Josef D. Järhult

**Affiliations:** a Department of Infectious Diseases, St. Jude Children’s Research Hospital, Memphis, Tennessee, USA; b Virology and Microbiology Research Group, School of Health Sciences, College of Health Sciences, University of KwaZulu-Natal, Westville Campus, Durban, South Africa; c Infectious Diseases and Anti-Infective Therapy Research Group, Sharjah Medical Research Institute and College of Pharmacy, University of Sharjah, Sharjah, United Arab Emirates; d Zoonosis Science Center, Department of Medical Sciences, Uppsala University, Uppsala, Sweden; e Institut Pasteur, National Reference Center & World Health Organization Collaborating Center for Listeria, Paris, France; f Institut Pasteur, Biology of Infection Unit, Paris, France; g Inserm U1117, Paris, France; h Université de Paris, Institut Imagine, Necker-Enfants Malades University Hospital, Division of Infectious Diseases and Tropical Medicine, APHP, Paris, France; i Discipline of Genetics, School of Life Sciences, College of Agriculture, Engineering and Science, University of KwaZulu-Natal, Durban, South Africa; j Department of Veterinary Population Medicine, College of Veterinary Medicine, University of Minnesota, St. Paul, Minnesota, USA; University of Rochester School of Medicine and Dentistry

## Abstract

Here, we report the draft genome sequence of Listeria innocua strain MEZLIS26, isolated from a healthy goat in Flagstaff, Eastern Cape Province, South Africa. The genome was sequenced using the Illumina MiSeq platform and had a length of 2,800,777 bp, with a G+C content of 37.4%, 2,755 coding DNA sequences (CDSs), 49 transfer RNAs (tRNAs), and 4 noncoding RNAs (ncRNAs).

## ANNOUNCEMENT

Listeria spp. are small, motile, catalase-positive, non-spore-forming, rod-shaped, Gram-positive bacteria. The genus *Listeria* is currently known to consist of 20 species (1), of which L. monocytogenes is an important foodborne human pathogen causing serious epidemics and sporadic listeriosis (2, 3). *Listeria* spp. have been isolated from a wide variety of sources, and L. innocua is reported to be more commonly isolated than L. monocytogenes (4). *L. innocua* is a nonpathogenic surrogate species that is closely related to L. monocytogenes. Recently, atypical hemolytic L. innocua was reported to be virulent and can actively cross the intestinal epithelium and spread systemically to the liver and spleen, albeit to a lesser degree than L. monocytogenes (5). In addition to its clinical relevance (5–8) and similarity to L. monocytogenes, the genomes of *L. innocua* provide important information that helps understand the pathogenicity of L. monocytogenes. Limited data about the genome sequence of *L. innocua* are available. Here, we report the draft genome sequence of *L. innocua* strain MEZLIS26, isolated from a goat in Flagstaff, Eastern Cape, South Africa, in May 2018. The sample was collected in 10 ml of 0.1% buffered peptone water and incubated for 24 hours. Following enrichment in *Listeria* broth (Oxoid, England), the sample was streaked onto *Listeria* selective agar (Oxoid, England) and incubated at 37°C for 18 hours. A slant of the bacterial culture was shipped to North Carolina State University (NCSU) for further analysis as part of the GenomeTrakr project (9).

Colony PCR for the hemolysin (*hly*) gene was performed as previously described (10). An aliquot of overnight culture in brain heart infusion (BHI) broth was submitted to the Clinical Sciences Department at NCSU for matrix-assisted laser desorption ionization–time of flight mass spectrometry (MALDI-TOF MS) analysis for further confirmation. DNA isolation was performed using a MasterPure DNA isolation kit (Lucigen, WI) according to the manufacturer’s protocol. Sequencing libraries were prepared using a Nextera XT library preparation kit (Illumina, CA). Sequencing was performed on the Illumina MiSeq platform using the v2 reagent kit, which yielded 250-bp paired-end (PE) reads.

**FIG 1:**
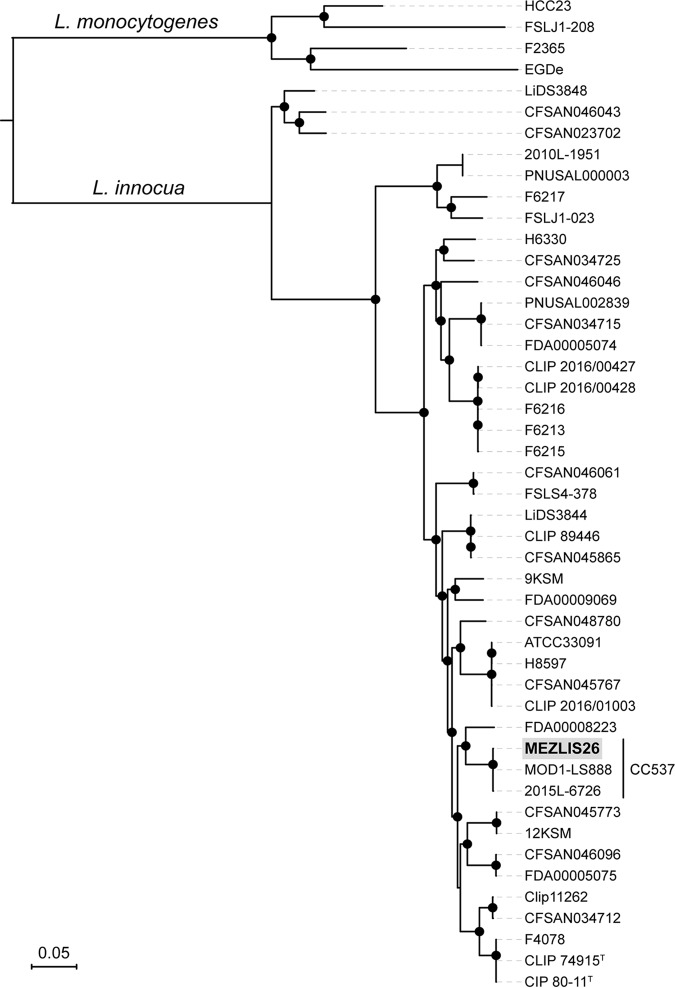
Phylogenetic positioning of isolate MEZLIS26 (highlighted in gray) within L. innocua. Representative genomes of L. monocytogenes were used as the outgroup. The maximum likelihood phylogeny was inferred from 642,408 core genome SNPs. Black circles represent bootstrap branch support values higher than 90% based on 1,000 replicates.

A total of 1,294 Mb (or ∼1.3 Gb) raw data reads were generated, and a total of 1.191 Mb (or ∼1.2 Gb) cleaned reads were obtained using Trim Galore, a Perl wrapper for Cutadapt (11), and FastQC (12) using the functions –paired, –phred33, –clip_R1 11, –clip_R2 11, –three_prime_clip_R1 3, and –three_prime_clip_R2 3. The *N*_50_ value of the cleaned sequence reads was 234 bp. Sequences were assembled using Unicycler version 0.4.7 (13) into 12 contigs of at least 200 nucleotides (nt) long, using default parameters with the addition of the –min_fasta_length 200 parameter. Assembly quality was assessed using QUAST (13), yielding a total of 2,800,777 bp, with a G+C content of 37.4%, an *N*_50_ value of 1,410,057 bp, and an *L*_50_ value of 1. Prokka version 1.13 (14) was used for annotation, indicating that the genome contained 2,755 coding DNA sequences (CDSs) and 49 tRNA, 1 transfer-messenger RNA (tmRNA), and 3 rRNA genes. The average nucleotide identity BLAST against L. innocua Clip11262 (GenBank accession number NC_003212) was of 98.73%, confirming species identity (15). To better understand the phylogenetic placement of isolate MEZLIS26, a maximum likelihood phylogeny was inferred from the core genome alignment of 42 L. innocua and 4 L. monocytogenes public genomes (5) using Parsnp, implemented in Harvest suite v.1.1.2 (16) and visualized with iTol v.4.2 (17). Isolate MEZLIS26 clustered within clonal complex CC537 (nonhemolytic L. innocua) together with isolates MOD1-LS888 and 2015L-6726 (SRA accession numbers SRR1481929 and SRR2915359, respectively), isolated from food in the United States (Fig. 1).

### Data availability.

This whole-genome sequencing project has been deposited at DDBJ/ENA/GenBank under the BioProject number PRJNA514279 (BioSample accession number SAMN11604718 and GenBank accession number AADHQU000000000). The version described in this paper is the first version, AADHQU010000000. The sequences have been submitted to the Sequence Read Archive (SRA) under the accession numbers SRX5806851 and SRR9029426. All isolates used in this study are also publicly available in https://bigsdb.pasteur.fr/listeria/.
